# Supplementary material for: Determining the maximum information gain and optimizing experimental design in neutron reflectometry using the Fisher information
Source: J Appl Crystallogr. 2021 Jul 7;54(Pt 4):1100–10. doi: 10.1107/S160057672100563X (PMC8366423; doi:10.1107/S160057672100563X)
Supplement: Supplementary file 1 [file j-54-01100-sup1.pdf]

JOURNAL OF APPLIED CRYSTALLOGRAPHY

Volume 54 (2021)

Supporting information for article:

Determining the maximum information gain and optimising experimental design in neutron reflectometry using the Fisher information

James H. Durant, Lucas Wilkins, Keith Butler and Joshaniel F. K. Cooper

Determining the maximum information gain and optimising experimental design in neutron reflectometry using the Fisher information

James H. Durant[1], Lucas Wilkins[2], Keith Butler[3], and Joshaniel F. K. Cooper[1]

[1]ISIS Neutron and Muon source, Rutherford Appleton Laboratory, Harwell Campus, OX11 0QX
[2]Department of Zoology, University of Oxford, Mansfield Road, Oxford, OX1 3SZ
[3]SciML, Scientific Computing Division, Rutherford Appleton Laboratory, Harwell Campus, OX11 0QX

1 Supplementary Information

1.1 Deriving the Fisher Information

1.1.1 Notation

N	Number of bins or equivalently, data points
M	Number of model parameters
λ_i	Expected neutron counts in bin (parameter) i
$\lambda = (\lambda_1 ... \lambda_N)$	Vector of expected neutron counts in all bins (parameter)
τ_θ	Time recording at experimental condition θ, e.g. angle, contrast
$\mu_{i\theta}$	Incident flux in bin i for condition θ (parameter)
g^z	Fisher information for parameterisation z
ξ_j	Parameters of the structure model
$r_i(\xi)$	Reflectivity for bin i from model with parameters ξ
X	Random variable describing a full measurement of N points
$x_i \in \mathcal{X}_i$	Neutron count in bin i
$p(x; z)$	Probability distribution for measurements x, parameterised by z
$\Pr([\text{event}])$	Probability of event
s_i	Total number of incident neutrons in bin i

1.1.2 The Model

In reflectometry, a model describes the reflectivity at a given neutron momentum transfer. These momentum transfer values are binned with the measured number of neutrons in bin i being given by

$$\lambda_i = r_i s_i(\tau) \tag{1}$$

where s_i is the number of neutrons incident in bin i. s_i is a function of the number of incident neutrons in the ith bin at each experimental condition, $\mu_{i\theta}$, and the time each condition is measured for τ_θ.

$$s_i(\tau) = \sum_k \tau_k \mu_{ik}$$

and so

$$\lambda_i(\xi) = \sum_\theta r_i(\xi)\tau_\theta \mu_{i\theta}$$

1.1.3 Fisher Information about the λ coordinates

The probability distribution of the measurement in one bin is Poisson distributed

$$\Pr(X_i = x_i; \lambda_i) = \frac{e^{-\lambda_i}\lambda_i^{x_i}}{x_i!}$$

and the corresponding Fisher information (FI) for this bin, with respect to λ_i, is

$$g^{\lambda_i} = 1/\lambda_i = 1/\mathbb{E}[\operatorname{var} X_i]$$

The probability distribution for the whole measurement is, by independence

$$\Pr(X = (x_0...x_N)) = \prod_i Pr(X_i = x_i)$$

and the corresponding FI with respect to λ is

$$g^\lambda_{jk} = \begin{cases} g^{\lambda_k} & \text{if } j = k \\ 0 & \text{otherwise} \end{cases}$$

i.e. a diagonal matrix with values of g^{λ_i} which happens to equal (in this case, but not in general), $\mathbb{E}[\operatorname{cov} X]^{-1}$.

1.1.4 Fisher Information about the ξ coordinates.

In general, we can transform the FI using tensor transforms, i.e.

$$g^Z_{ij} = g^Y_{ab}\frac{\partial y_a}{\partial z_i}\frac{\partial y_b}{\partial z_j}$$

So, the FI in terms of ξ is just

$$g^\xi_{ij} = g^\lambda_{ab}\frac{\partial \lambda_a}{\partial \xi_i}\frac{\partial \lambda_b}{\partial \xi_j}$$

To get the FI as a function of τ, we take the derivative of equation 1 with respect to ξ which gives us

$$\frac{\partial \lambda_i}{\partial \xi_j} = s_i(\tau)\frac{\partial r_i}{\partial \xi_j} \tag{2}$$

The derivative $\frac{\partial r_i}{\partial \xi_j}$ is obtained from the model alone (irrespective of the data); it is the derivative of the reflectivity for bin i with respect to the jth ξ parameter.

We can now put everything together. First, we have the initial FI about the λ parameter, which we can write in terms of s_i and r_i

$$\mathbf{g}^{\lambda} = \begin{bmatrix} 1/s_1 r_1 & 0 & \cdots & 0 \\ 0 & 1/s_2 r_2 & \cdots & 0 \\ \vdots & \vdots & \ddots & \vdots \\ 0 & 0 & \cdots & 1/s_N r_N \end{bmatrix}$$

Equation 2 can be re-written in matrix form in terms of a diagonal matrix, $\mathbf{S}$, and the Jacobian matrix, $\mathbf{J}$, for the N modelled reflectivity points, with respect to the M parameters, ξ.

$$\mathbf{S} = \begin{bmatrix} s_1 & 0 & \cdots & 0 \\ 0 & s_2 & \cdots & 0 \\ \vdots & \vdots & \ddots & \vdots \\ 0 & 0 & \cdots & s_N \end{bmatrix}$$

$$\mathbf{J} = \begin{bmatrix} \frac{\partial r_1}{\partial \xi_1} & \frac{\partial r_1}{\partial \xi_2} & \cdots & \frac{\partial r_1}{\partial \xi_M} \\ \frac{\partial r_2}{\partial \xi_1} & \frac{\partial r_2}{\partial \xi_2} & \cdots & \frac{\partial r_2}{\partial \xi_M} \\ \vdots & \vdots & \ddots & \vdots \\ \frac{\partial r_N}{\partial \xi_1} & \frac{\partial r_N}{\partial \xi_2} & \cdots & \frac{\partial r_N}{\partial \xi_M} \end{bmatrix}$$

The tensor transformation of $\mathbf{g}^{\lambda}$ in this notation is then

$$\mathbf{g}^{\xi} = (\mathbf{SJ})^T \mathbf{g}^{\lambda} (\mathbf{SJ}) = \mathbf{J}^T \mathbf{S} \mathbf{g}^{\lambda} \mathbf{S} \mathbf{J}$$

$$(M \times M) = (M \times N)(N \times N)(N \times N)(N \times N)(N \times M)$$

and the matrix $\mathbf{S}\mathbf{g}^{\lambda}\mathbf{S}$ is a composition of diagonal matrices, and is equal to

$$\begin{bmatrix} s_1/r_1 & 0 & \cdots & 0 \\ 0 & s_2/r_2 & \cdots & 0 \\ \vdots & \vdots & \ddots & \vdots \\ 0 & 0 & \cdots & s_N/r_N \end{bmatrix}$$

1.1.5 Summary

In summary, the FI about ξ is given by

$$\mathbf{g}^{\xi} = \mathbf{J}^{\mathbf{T}} \mathbf{M} \mathbf{J}$$

where, $\mathbf{J}$ is the Jacobian of the reflectances, r_i, with respect to the parameters, ξ. $\mathbf{M}$ is a diagonal matrix with entries $(s_0/r_0, s_1/r_1 \ldots s_N/r_N)$ and s_i is the incident neutron flux, which depends on the experimental condition k and the time spent measuring it, τ_k

$$s_i(\tau) = \sum_k \tau_k \mu_{ik}$$

1.2 Confidence Ellipses

To calculate confidence intervals in the general case, we can find the set of parameters that differ from the estimate by a certain number of standard deviations. To do this, we need to know the length of a vector in parameter space in terms of the number of standard deviations. This is what the FI does (technically, it gives a linear approximation to the informational distance between distributions, but they are related). So, if we want to find a vector with a given length, k, we solve

$$k^2 = \Delta\xi^T \mathbf{g} \Delta\xi$$

It so happens that k in the above equation can be interpreted as "number of standard deviations", so a 2σ error bar will have $k = 2$. In practice, it is useful to fix a direction, and calculate the magnitude of the vector needed to reach the threshold. I.e. let $\Delta\xi = \epsilon\widehat{\Delta\xi}$ where $\widehat{\text{hat}}$ denotes a unit vector. Consider the 1-D case; $k^2 = \epsilon^2 g$, g is analogous to the inverse variance, so we have $k^2 = \epsilon^2/\sigma^2$. Therefore, if we want to know where $\epsilon = 2\sigma$, we would have $k^2 = (2\sigma)^2/\sigma^2$ and thus, $k = 2$.

In 2-D, the unit vectors can be written as $(\sin\vartheta, \cos\vartheta)$. We can graphically solve the following

$$k^2 = \epsilon^2 \underbrace{\left(\begin{bmatrix} \sin\vartheta, \cos\vartheta \end{bmatrix} \mathbf{g}^\xi \begin{bmatrix} \sin\vartheta \\ \cos\vartheta \end{bmatrix} \right)}_{\text{scalar}}$$

for ϵ over a sample of angles, θ, in $[0, 2\pi]$ by plotting the points $(\epsilon(\theta)\sin\theta, \epsilon(\theta)\cos\theta)$. The result is a confidence ellipse of size k between two chosen parameters. If $|\xi| > 2$, then for two chosen parameters, ξ_i and ξ_j, the above equation becomes

$$k^2 = \epsilon^2 \left(\begin{bmatrix} \sin\vartheta, \cos\vartheta \end{bmatrix} \begin{bmatrix} \mathbf{g}^\xi_{i,i} & \mathbf{g}^\xi_{i,j} \\ \mathbf{g}^\xi_{j,i} & \mathbf{g}^\xi_{j,j} \end{bmatrix} \begin{bmatrix} \sin\vartheta \\ \cos\vartheta \end{bmatrix} \right)$$

1.3 Point Estimates vs Posterior Distributions

An estimator, usually written with a $\widehat{\text{hat}}$, is a function of sampled data that provides an estimate of a parameter. In frequentist statistics, one is concerned with the probability distributions of *estimators*, not of the parameters themselves (as would be the case in Bayesian statistics). The distribution of estimators often has a variance. When it does, and when it is unbiased, i.e.

$$\mathbb{E}[\hat{\xi}] = \xi$$

then it is related to the FI by the Cramér-Rao bound:

$$\operatorname{var}\hat{\xi} \succcurlyeq \frac{1}{N}(\mathbf{g}^\xi)^{-1}$$

1.4 Time Dependence

If there is a single measurement condition, we have $s_i = \mu_i\tau$. Then, we see that τ is a factor of all the s_i's and so of the matrix $\mathbf{M}$ and thus of $\mathbf{g}^\xi$. We can therefore write $\mathbf{g}^\xi = \tau\mathbf{f}^\xi$. The size of an

"error bar", ϵ, for a single parameter, ξ, at a threshold, k, in a direction, $\widehat{\Delta\xi}$, is given by

$$k^2 = (\epsilon\widehat{\Delta\xi})\mathbf{g}^{\xi}(\epsilon\widehat{\Delta\xi})$$

And so,

$$k^2 = (\epsilon\widehat{\Delta\xi})\tau\mathbf{f}^{\xi}(\epsilon\widehat{\Delta\xi})$$

Hence $\epsilon \propto 1/\sqrt{\tau}$.

1.5 Bilayer Model Parameterisation

For the measured DMPC sample, each experimental dataset was recorded with an instrument resolution of 2%. The instrument backgrounds were 3.21×10^{-6}, 2.80×10^{-6} and 2.06×10^{-6} respectively. The SLDs of the Si substrate and following SiO_2 layer were defined using known values of $2.073 \times 10^{-6}\text{Å}^{-2}$ and $3.41 \times 10^{-6}\text{Å}^{-2}$. From fitting in `RasCAL`, we obtained values of 14.7Å and 24.5% for the SiO2 layer's thickness and hydration. For both the Si/SiO_2 and SiO_2/DMPC interfacial roughnesses, we obtained 2.00Å; all other interfacial roughnesses shared a common parameter that was fitted as 6.57Å.

We have assumed the molecular volumes of the headgroups and tailgroups are known and constant at 320.9Å^3 and 783.3Å^3, and that any changes in molecule surface area are inversely proportional to the headgroup and tailgroup thicknesses. Therefore, we need only fit one parameter: the area per molecule (APM) at the surface. From the APM we can calculate the tailgroups thickness using the known volume.

$$\text{Thickness} = \frac{\text{Volume}}{\text{APM}} \tag{3}$$

From fitting the APM parameter, we obtained a value of 49.9Å^2. Also using the tailgroup volume, the SLD of the tailgroups can be calculated using the known tailgroup scattering length (SL) of -3.08×10^{-4}Å and equation

$$\text{SLD}(\rho) = \frac{\Sigma b}{\text{Volume}} \tag{4}$$

Both the headgroups and tailgroups contain water through defects across their surfaces but there is also water bound to the hydrophilic headgroups. The model accounted for these differing hydration types by varying two parameters: the total bilayer hydration and headgroup bound waters for which we obtained values of 7.37% and 3.59. The headgroup water SLs in H_2O and D_2O were calculated as the product of the bound waters parameter and the known SLs of -1.64×10^{-5}Å and 2.00×10^{-4}Å for the H_2O and D_2O solutions. Further, the product of the bound waters parameter and the known volume of water, 30.4Å^3, yielded the headgroup water volume. The headgroup thickness was calculated using the total headgroup volume (including bound water), APM parameter and equation 3. To determine the headgroup SLs in H_2O and D_2O we took the sum of the known headgroup SL of 6.41×10^{-4}Å and headgroup SL in H_2O and D_2O previously calculated. Finally, using the headgroup SL in each contrast and the calculated headgroup volume, we calculated the SLD of the headgroup in each solution using equation 4.

We reparamaterised the fitted model as a function of bulk water contrast SLD using the known SLD of the DMPC headgroups of $1.98 \times 10^{-6}\text{Å}^{-2}$ (if no hydrating water was present) and approximating the headgroup hydration at 27%.
